# Antiprotozoal and antimycobacterial activities of *Persea americana* seeds

**DOI:** 10.1186/1472-6882-13-109

**Published:** 2013-05-16

**Authors:** Adelina Jiménez-Arellanes, Julieta Luna-Herrera, Ricardo Ruiz-Nicolás, Jorge Cornejo-Garrido, Amparo Tapia, Lilián Yépez-Mulia

**Affiliations:** 1 Unidad de Investigación Médica en Farmacología (UIM), Hospital de Especialidades, CMNS XXI, Instituto Mexicano del Seguro Social (IMSS), Av. Cuauhtémoc 330, Col. Doctores, México, DF, 06720, Mexico; 2 Departamento de Inmunología, Escuela Nacional de Ciencias Biológicas, Instituto Politécnico Nacional, Plan de Ayala s/n, Col. Casco de Santo Tomás, México, DF, 11340, Mexico; 3 UIM en Enfermedades Infecciosas y Parasitarias, Hospital de Pediatría, 2° piso, CMNS XXI, IMSS, Av. Cuauhtémoc 330, Col. Doctores, México, DF, 06720, Mexico

**Keywords:** Medicinal plant, Seeds, Antimycobacterial, Antiprotozoal, *Persea americana*

## Abstract

**Background:**

*Persea americana* seeds are widely used in traditional Mexican medicine to treat rheumatism, asthma, infectious processes as well as diarrhea and dysentery caused by intestinal parasites.

**Methods:**

The chloroformic and ethanolic extracts of *P. americana* seeds were prepared by maceration and their amoebicidal, giardicidal and trichomonicidal activity was evaluated. These extracts were also tested against *Mycobacterium tuberculosis* H37Rv, four mono-resistant and two multidrug resistant strains of *M. tuberculosis* as well as five non tuberculosis mycobacterium strains by MABA assay.

**Results:**

The chloroformic and ethanolic extracts of *P. americana* seeds showed significant activity against *E. histolytica, G. lamblia* and *T. vaginalis* (IC_50_ <0.634 μg/ml)*.* The chloroformic extract inhibited the growth of *M. tuberculosis* H37Rv, *M. tuberculosis* MDR SIN 4 isolate, three *M. tuberculosis* H37Rv mono-resistant reference strains and four non tuberculosis mycobacteria (*M. fortuitum, M. avium, M. smegmatis* and *M. absessus*) showing MIC values ≤50 μg/ml. Contrariwise, the ethanolic extract affected only the growth of two mono-resistant strains of *M. tuberculosis* H37Rv and *M. smegmatis* (MIC ≤50 μg/ml).

**Conclusions:**

The CHCl_3_ and EtOH seed extracts from *P. americana* showed amoebicidal and giardicidal activity. Importantly, the CHCl_3_ extract inhibited the growth of a MDR *M. tuberculosis* isolate and three out of four mono-resistant reference strains of *M. tuberculosis* H37Rv, showing a MIC = 50 μg/ml. This extract was also active against the NTM strains, *M. fortuitum, M. avium, M. smegmatis* and *M. abscessus,* with MIC values <50 μg/ml.

## Background

*Persea americana* Mill. (Lauraceae) is an edible fruit commonly known as *aguacate* (avocado) that grows throughout the tropics. The seeds (crude or toasted) are employed in traditional Mexican medicine to treat skin rashes, diarrhea, and dysentery caused by helminths and amoebas, for the cure of infectious processes caused by fungi and bacteria, as well as for the treatment of asthma, high blood pressure, and rheumatism [1-5]. The seeds of *P. americana* used alone or mixed with other species, such as *Psidium guajava, Mentha piperita* or *Ocimum basilicum*, are mainly employed for the treatment of diarrhea [4]*.*

The presence of fatty acids (linoleic, oleic, palmitic, stearic, linolenic, capric and myristic acids), polyphenols (catechin, isocatechin, protocyanidin, flavonoids, tannins and proanthocyanidin monomerics), saponins, glucosides (D-perseit, D-α-manoheptit, D-monoheptulose, persiteol), sterols (β-sitosterol, campesterol, stigmasterol, cholesterol), the amino acid carnitine and two glucosides of abscicic acid has been described for *P. americana* seeds [4-8]. High concentrations of catechins, procyanidins and hydroxycinnamic acid have recently been determined in 100% ethyl acetate (EtOAc), in 70% acetone and 70% methanol (MeOH) extracts obtained from *P. americana* peel and seeds, while the pulp extract was rich in hydroxybenzoic acid, hydroxycinnamic acid and procyanidins [9].

Interestingly, the hypolipemic effect of the MeOH extract obtained from *P. americana* seeds has been demonstrated in male rats with induced hypercholesterolemia [10,11]. This extract reduced total cholesterol levels, triglycerides and Low density lipoprotein (LDL); on the other hand, increased the levels of High density lipoprotein (HDL). The same effect was described for the aqueous extract, which also reduced blood pressure both in normal rats and those with high blood pressure; in addition, it exerted a hypoglycemic effect on rats and rabbits with diabetes [12-15]. The aqueous extract showed a median Lethal dose (LD_50_) = 10 g/kg in rats when it was administered orally. Importantly, it did not alter the hematological parameters nor the levels of Alanine aminotransferase (ALT), Aspartate aminotransferase (AST), albumin, and creatinine in male and female rats were treated for 28 days [16].

The hexanic and MeOH seed extracts of *P. americana* have been described to have a Minimum inhibitory concentration (MIC) of <1.25 μg/ml against *Candida* ssp.*, Cryptococcus neoformans* and *Malassezia pachydermatis.* These extracts were also active against *Artemia salina*, with Lethal concentration (LC_50_) values of 2.37 and 24.13 mg/ml, respectively. They were also active against *Aedes aegypti* larvae with LC_50_ values of 16.7 and 8.9 mg/ml, respectively [17]. On the other hand, the MeOH extract from *P. americana* leaves inhibited completely the growth of *M. tuberculosis* H37Ra (MIC = 125 μg/ml) and H37Rv (MIC = 62.5 μg/ml); furthermore, the hexane fraction inhibited the growth of both mycobacteria with MIC = 31.2 μg/ml [18]. In addition, the EtOH extract was active against both Gram-positive and -negative bacteria (with the exception of *Staphylococcus epidermis* and *Escherichia coli*) with MIC = 500 μg/ml [19]. Regarding the bacterial activity of *P. americana* (var Hass and Fuerte), the acetone seed extract exhibited moderate activity against *Bacillus cereus, Staphylococcus aureus* and *Listeria monocytogenes*[9]. The trypanomicidal activity of the MeOH extract from *P. americana* seeds has been also tested [20]. It showed moderate activity when was evaluated at the concentration range of 250–500 μg/ml. In the case of the aqueous seed extract, it had a slight anti-*Giardia duodenalis* (syn *G. lamblia*) activity, inducing 23% of mortality at 4 mg/ml [21].

An important antioxidant activity (AOA) of the MeOH extract of *P. americana* seeds and leaves has been described by different methods [8,22-24]. Besides, AOA has been reported in the 100% EtOAc, 70% acetone and 70% MeOH of the peel, pulp and seed extract [9].

Up to now, the activity of the chloroformic (CHCl_3_) and ethanol (EtOH) extracts obtained from *P. americana* seeds against anaerobic protozoan and *M. tuberculosis* H37Rv strains with different level of drug resistance has not yet been evaluated. Therefore, herein, the activity of both extracts was tested against the anaerobic protozoa *Giardia lamblia*, *Entamoeba histolytica* and *Trichomonas vaginalis*. In addition, their antimycobacterial activity was evaluated against four mono-resistant reference strains of *M. tuberculosis* H37Rv, two MDR *M. tuberculosis* clinical isolates and five non-tuberculosis mycobacterium (NTM)*.*

## Methods

### Plant material

*P. americana* seeds were obtained from the town of Ario de Rosales in the state of Michoacan, Mexico in August 2009. This material was then dried at room temperature and under conditions of darkness; the material was then ground. The plant was botanically identified by Abigail Aguilar, M.Sc., and a voucher specimen was deposited at the Herbarium of the Instituto Mexicano del Seguro Social, Mexico (IMSSM) with code number 14256.

### Preparation of extracts

The dry and powdered plant material (1.364 kg) was macerated three times with CHCl_3_ analytical reagent -AR- (J.T. Baker) at room temperature for 7 days. The extract was filtered and concentrated to dryness under low pressure at 40°C. The plant material was later macerated with EtOH AR grade (J.T. Baker) three times for 7 days and the solvent was eliminated under reduced pressure until the solvent-free extract was obtained.

### Antiprotozoal activity evaluation

For this assay, the *E. histolytica* strain HM1-IMSS and the *T. vaginalis* GT9 strain were cultured in a TYI-S-33 modified medium supplemented with 10% calf serum; *G. lamblia* strain IMSS:0989:1 was maintained in a TYI-S-33 medium supplemented with 10% calf serum and bovine bile. *In vitro* susceptibility assays were performed according to the method previously described [25,26]. Briefly, 5 × 10^4^ trophozoites of *G. lamblia* were incubated for 48 h at 37°C with increasing concentrations of the EtOH and CHCl_3_ extracts of *P. americana* seeds using Dimethyl sulfoxide (DMSO) as a suitable solvent. After incubation, *G. lamblia* trophozoites were washed and subcultured for an additional 48 h in a fresh medium alone. For *E. histolytica* and *T. vaginalis,* 6 × 10^3^ trophozoites were incubated for 72 h at 37°C with increasing concentrations of the samples tested. Metronidazole was included as a positive control; parasites without treatment but with the highest DMSO concentration used for sample dilutions were included as a negative control. *G. lamblia, T. vaginalis* and *E. histolytica* trophozoites were counted and the 50% Inhibitory concentration (IC_50_) was calculated by Probit analysis. The experiments were carried out in triplicate and repeated at least twice.

### Antimycobacterial evaluation

For this assay, 12 Mycobacterium strains were employed: *M. tuberculosis* H37Rv (ATCC 27294, a strain sensitive to streptomycin (STR), isoniazid (INH), rifampicin (RIF), etambutol (EMB), or pyrazinamide; *M. tuberculosis* SIN 4 (a MDR clinical isolate with resistance to first-line drugs); *M. tuberculosis* MMDO (an MDR clinical isolate with resistance to INH and EMB); four mono-resistant strains of *M. tuberculosis* H37Rv (INH-R, ATCC 35822; STR-R, ATCC 35820; RIF-R, ATCC 35838, and EMB-R, ATCC 35837) and four NTM clinical isolates (*M. fortuitum*, *M. chelonae*, *M. abscessus* and *M. avium)* and *M. smegmatis* (ATCC 35798). The Mycobacterium strains were grown and maintained in Middlebrook 7H9 broth supplemented with 10% OADC enrichment (Becton Dickenson, USA) at 37°C until a logarithmic growth phase was achieved. At the moment of evaluation, *M. tuberculosis* strains and NTM were diluted 1:20 and 1:50, respectively in the 7H9 medium.

The antimycobacterial activity was carried out by means of the Microplate alamar blue assay (MABA) previously described [26,27]. EtOH and CHCl_3_ extracts (10 mg) were solubilized in 500 μL of DMSO and from these stock solutions, several dilutions were prepared to achieve concentrations between 200 and 3.13 μg/ml. The maximum DMSO concentration used in the MABA assay does not affect mycobacterial growth, as it was previously reported [28]. Extracts that presented MIC <100 μg/ml were considered to have good antimycobacterial activity. Rifampicin and isoniazid at 0.06 μg/mL were included as a positive control for the H37Rv strain, for the MDR clinical isolates and for NTM; the same drugs were used but at concentrations of 100 and 3.1 μg/ml, respectively.

## Results and discussion

By means of the maceration process, 88.7 g of the CHCl_3_ extract and 77.2 g of the EtOH extract from *P. americana* seeds were obtained with an average yield of 6% with respect to the plant material’s dry weight. A preliminary phytochemical analysis by Thin layer chromatography (TLC) of the CHCl_3_ extract led to the detection of the presence of β-sitosterol, phytol and palmitic acid. On the other hand, catechin and epicatechin were detected in the EtOH extract by TLC. All compounds were identified by comparison of the Retention factor (R_*f*_) with their commercial reference. A previous study by Rodríguez-Carpena et al. [9] reported a high concentration of catechin and epicatechin in 100% EtOAc, 70% acetone and 70% MeOH extracts obtained from *P. americana* seeds and peel.

The EtOH and CHCl_3_ extracts displayed significant antiprotozoal activity. For *G. lamblia, E. histolytica* and *T. vaginalis*, the CHCl_3_ extract showed values of IC_50_ = 0.634, 0.417 and 0.524 μg/ml, respectively. On the other hand, the EtOH extract exhibited IC_50_ values of 0.486, 0.386 and 0.533 μg/ml against *G. lamblia*, *E*_*.*_*histolytica* and *T. vaginalis*, respectively (Table 1)*.* Although the giardicidal activity of the aqueous seed extract has been previously evaluated [21], the authors used the 3-(4,5-dimetiltiazol-2-ilo)-2,5-difeniltetrazol (MTT) colorimetric method; therefore our data can not be compared.

**Table 1:** **Antiprotozoal activity of the CHCl**_**3**_**and EtOH extracts from*Persea americana*seeds**

**Sample**	**IC**_**50**_**(μg/ml)**		
	***G. lamblia***	***E. histolytica***	***T. vaginalis***
CHCl_3_ extract	0.634	0.417	0.524
EtOH extract	0.486	0.386	0.533
Metronidazole	0.210	0.06	0.037

*IC*_*50*_*: medium Inhibitory concentration.*

It is interesting to note that, the reference drug, metronidazole (IC_50_ = 0.210 μg/ml) showed only three and two times higher anti-*Giardia* activity than the CHCl_3_ and EtOH extracts. However, in the case of *E. histolytica*, the CHCl_3_ and EtOH extracts were seven and six times less potent than metronidazole (IC_50_ = 0.060 μg/ml). For *T. vaginalis*, these extracts showed weak activity, being 16 (CHCl_3_) and 17 (EtOH) times less potent than metronidazole (CI_50_ = 0.037 μg/ml).

In traditional Mexican medicine, *P. americana* seeds are used to treat diarrhea [1-4]. It is shown here that *P. americana* EtOH and CHCl_3_ seed extracts are indeed responsible for the activity against two anaerobic parasites that cause diarrhea: *G. lamblia*, and *E. histolytica*. The *in vitro* and *in vivo* giardicidal activity of epicatechin isolated from plants such as *Rubus coriifolius* and *Heliantemum glomeratus* has been reported [29,30]. In this regard, epicatechin was detected in *P. americana* EtOH seed extract by TLC analysis; hence, this compound may be responsible for the giardicidal activity observed in this study. Further studies are required to define the active compound(s) responsible for the antiprotozoal activity of the CHCl_3_ extract.

It is worth considering the fact that the EtOH and CHCl_3_ extracts constitute potential sources of compounds that can be employed as prototype molecules for the development of novel antiprotozoal agents as an alternative treatment of clinical isolates with metronidazole resistance.

The results of the antimycobacterial activity of the EtOH and CHCl_3_ extracts determined by MABA assay are presented in Table 2. It is important to note that the CHCl_3_ extract inhibited the growth of *M. tuberculosis* H37Rv, MDR *M. tuberculosis* SIN 4 and three out of four mono-resistant reference strains of *M. tuberculosis* H37Rv (INH-R, STR-R, and EMB-R), showing a MIC = 50 μg/ml. This extract was also active against the NTM: *M. fortuitum, M. avium, M. smegmatis* and *M. abscessus* with MIC values < 50 μg/ml. However, the EtOH extract affected only the growth of *M. smegmatis* (MIC = 25 μg/ml) and the mono-resistant strains of *M. tuberculosis* H37Rv STR-R and EMB-R (MIC = 50 μg/ml).

**Table 2:** **Antimycobacterial effect of the CHCl**_**3**_**and EtOH extracts from*Persea americana*seeds**

***Mycobacterium tuberculosis***	**Drug-resistance pattern**	**MIC (μg/ml)**	
		**CHCl**_**3**_**extract**	**EtOH extract**
H37Rv	INH-, RIF-, STR-, and EMB- susceptible	50	>100
Clinical isolates			
SIN4	STR, INH, RIF, EMB, RFB, ETH, and OFX	50	>100
MMDO	INH, EMB	100	>100
Mono-resistant			
RIF-R	RIF	>100	>100
INH-R	INH	50	>100
STR-R	STR	50	50
EMB-R	EMB	50	50
NTB *Mycobacterium*			
*M. fortuitum*		50	100
*M. avium*		25	100
*M. chelonae*		100	100
*M. smegmatis*		12.5	25
*M. abscessus*		25	100

SIN 4 MDR clinical isolate of *M. tuberculosis* resistant to STR: Streptomycin, INH: Isoniazid, RIF: Rifampicin, EMB: Ethambutol, RFB: Rifabutin, ETH: Ethionamide and OFX: Ofloxacin. MMDO MDR clinical isolates of *M. tuberculosis* resistant to INH and EMB; the resistance pattern was determined by Microdilution alamar blue assay (MABA). NTB: non-tuberculosis mycobacteria; MIC: Minimum inhibitory concentration. Data are means of three determinations.

To date, the hexanic and MeOH extracts obtained from the stems and leaves of *P. americana* have been reported to inhibit the growth of *M. tuberculosis* H37Rv and *M. tuberculosis* H37Ra strains [18,31]. The reported antimycobacterial activity of the MeOH extracts was attributed to the presence of lignans [31] and the MIC was slightly lower than the CHCl_3_ extract tested in this study.

Since the presence of MDR and extensively drug-resistant (XDR) *M. tuberculosis* cases is increasing rapidly and current chemotherapy is prolonged, poorly effective, expensive and is accompanied by severe side effects [26]; it is necessary to have recourse to an alternative treatment against these strains or that can even aid in and/or shorten the currently available that could have a different mechanism of action. Therefore, the high activity exhibited by the *P. americana* CHCl_3_ seed extract against mono-resistant strains of *M. tuberculosis* H37Rv, MDR clinical isolates and NTM that is described in this study, it is of great interest.

From the clinical point of view, NTM are becoming relevant, because of the so-called mycobacteriosis and are currently recognized as important pathogens associated with both immune-deficient and immune-competent patients. The mycobacteria tested in this study are representative of the most common NTM isolated from pulmonary cases (*M. abscessus* and *M. avium*) or associated with soft tissue infections *(M. fortuitum* and *M. chelonae*) [32]. Although *M. smegmatis* is a poor pathogenic bacterium, it was included in the NTM group because it is widely employed in the determination of the antimycobacterial activity of new compounds [32,33]. In the case of NTM, the majority is naturally resistant to some of the first-line anti-TB drugs such as INH and RIF; thus, effective drugs against NTM are scarce than those for TB, emphasizing the urgency of finding novel active compounds that could be used in the treatment of the NTM group. Based on our results, *P. americana* seeds may be a source for potential moieties (molecules) against NTM. We are currently conducting the isolation and identification of the active compounds responsible for the antimycobacterial activity observed with the CHCl_3_ extract.

## Conclusions

Herein, to the best of our knowledge, the activity of the CHCl_3_ and EtOH seed extracts from *P. americana* against two intestinal parasites that cause diarrhea: *E. histolytica*, and *G. lamblia* has been demonstrated. In addition, based on our results, CHCl_3_ seed extract may be a source for potential moieties (molecules) against *M. tuberculosis* drug-resistant species as well as NTM.

Further studies are required for the identification of the active compounds responsible for the antiprotozoal and antimycobacterial activity observed with the EtOH and CHCl_3_ seed extracts from *P. americana*.

## Abbreviations

ALT: Alanine aminotransferase; AST: Aspartate aminotransferase; AOA: Antioxidant activity; CHCl3: Chloroformic; AR: Analytical reagent; DMSO: Dimethyl sulfoxide; EtOAc: Ethyl acetate; EtOH: Ethanol; MeOH: Methanol; MTT: 3-(4,5-dimetiltiazol-2-ilo)-2,5-difeniltetrazol; IMSSM: Instituto Mexicano del Seguro Social Mexico; P. americana: Persea americana; HDL: High density lipoprotein; LDL: Low density lipoprotein; Rf: Retention factor; TLC: Thin layer chromatography; EMB: Etambutol; INH: Isoniazid; STR: Streptomycin; RIF: Rifampicin; LC50: Lethal concentration; LD50: Lethal dose; MIC: Minimum inhibitory concentration; IC50: Medium Inhibitory concentration; MABA: Microplate alamar blue assay; MDR: Multidrug-resistant; XDR: Extensively drug-resistant; M. fortuitum: *Mycobacterium fotuitum*; M. avium: *Mycobacterium avium*; M. smegmatis: *Mycobacterium smegmatis*; M. absessus: *Mycobacterium absessus*; M. tuberculosis: *Mycobacterium tuberculosis*; NTM: *non-*tuberculosis mycobacterium; E. histolytica: *Entamoeba histolytica*; G. lamblia: *Giardia lamblia*; T. vaginalis: *Trichomonas vaginalis*.

## Competing interests

The authors declare that they have no competing interests.

## Authors’ contributions

AJ-A designed and coordinated the study, prepared the CHCl_3_ and EtOH extracts and carried out their phytochemical analysis and wrote the manuscript. C-G collected the *P. americana* seeds and contributed to the preparing the CHCl_3_ and EtOH extracts. JL-H and RR-N evaluated the antimycobacterial activity from CHCl_3_ and EtOH extracts. The antiprotozoal activity from both extracts was determined by AT and LY-M, who also contributed to the manuscript preparation. All authors have read and approved the final manuscript.

## Pre-publication history

The pre-publication history for this paper can be accessed here:

http://www.biomedcentral.com/1472-6882/13/109/prepub
